# The Level of IgA Antibodies to Endothelial Cells Correlates with Histological Evidence of Disease Activity in Patients with Lupus Nephritis

**DOI:** 10.1371/journal.pone.0163085

**Published:** 2016-10-27

**Authors:** Ayako Kondo, Kazuo Takahashi, Tomohiro Mizuno, Akihiro Kato, Daisuke Hirano, Naoki Yamamoto, Hiroki Hayashi, Shigehisa Koide, Hiroshi Takahashi, Midori Hasegawa, Yoshiyuki Hiki, Shunji Yoshida, Keiji Miura, Yukio Yuzawa

**Affiliations:** 1 Department of Nephrology, Fujita Health University School of Medicine, Toyoake, Aichi, Japan; 2 Analytical Pharmacology, Faculty of Pharmacy, Meijo University, Nagoya, Aichi, Japan; 3 Department of Rheumatology, Fujita Health University School of Medicine, Toyoake, Aichi, Japan; 4 Institute of Joint Research, Fujita Health University, Toyoake, Aichi, Japan; 5 Fujita Health University School of Health Sciences, Toyoake, Aichi, Japan; Nippon Medical School, JAPAN

## Abstract

Anti-endothelial cell antibodies (AECA) are frequently detected in patients with systemic lupus erythematosus (SLE), but their pathological role remains unclear. We recently developed a solubilized cell surface protein capture enzyme-linked immunosorbent assay (CSP-ELISA) to detect antibodies against membrane proteins involved in autoimmune reactions. In this study, sera from 51 patients with biopsy-proven lupus nephritis (LN), 25 with SLE without renal involvement (non-LN SLE), 42 disease control (DC) subjects, and 80 healthy control (HC) subjects were tested for IgG- and IgA-AECA for human umbilical vein endothelial cells (HUVEC) and human glomerular EC (HGEC) by using CSP-ELISA. IgG- and IgA-AECA titers were significantly higher in LN and non-LN SLE patients than in the DC or HC (*P* < 0.001) groups. IgG- and IgA-AECA titers for HUVEC corresponded well with those for HGEC. The IgA-AECA level correlated with the SLE disease activity index and with histological evidence of active lesions (cellular proliferations, hyaline thrombi and wire loops, leukocytic infiltration, and fibrinoid necrosis) in LN patients (*P* < 0.001). The sensitivity of IgA-AECA as a diagnostic test for histological evidence of active lesions in LN patients was 0.92, with a specificity of 0.70. The significant correlation of IgA-AECA with glomerular hypercellularity indicates that IgA-AECA are associated with endothelial damage in LN.

## Introduction

Systemic lupus erythematosus (SLE) is a systemic autoimmune disease affecting various tissues, with diverse clinical manifestations accompanied by the presence of numerous autoantibodies. In contrast to other classical autoimmune diseases, the autoantigens in SLE are still under investigation [1]. This lack of knowledge precludes the development of precise diagnostic tools, and precise and causal therapeutic interventions [2].

Lupus nephritis (LN) is one of the most serious manifestations of SLE and a predictor of poor renal outcomes and overall survival of SLE patients [3]. The spectrum of kidney lesions in SLE patients is wide and the mechanisms leading to kidney inflammation are not completely elucidated; however, autoantibodies seem to play a pivotal role. Renal biopsy is the gold standard for providing information on histological classes of LN and the relative degree of disease activity [4]. The morphologic lesions range from minimal mesangial alterations to severe immune complex deposition with proliferative lesions and necrosis, and the current management for LN is based upon renal histology class [5]. To improve the efficacy and decrease the adverse effects of immunosuppression, determination of the pathology of LN and appropriate adjustment of therapy are needed. Thus, biomarkers that reflect the activity of LN are required [6].

Anti-endothelial cell antibodies (AECA) represent a heterogeneous group of antibodies against poorly characterized targets. AECA have been reported in a wide variety of systemic disorders associated with vascular injury including SLE, systemic sclerosis, mixed connective tissue disease, Takayasu’s arteritis, granulomatosis with polyangiitis, Behcet’s disease, and transplant arteriosclerosis, and they may be valuable as markers of disease activity [7–9]. AECA have been reported to cause endothelial dysfunction, and recognize a diverse spectrum of antigens on endothelial cells as demonstrated by in vitro studies with human umbilical vein endothelial cells (HUVEC) and endothelial cells of other tissues [10–12]. AECA are commonly immunoglobulin (Ig) G, but IgA- and IgM-AECA have also been described, such as IgA-type AECA in IgA nephropathy and Henoch–Schönlein purpura nephritis [13, 14]. Although the role of IgA-AECA in SLE has not been well described, IgA-AECA may play an important role in LN as not only anti-DNA IgG but also anti-DNA IgA is associated with both LN and active disease [15–17].

Because antigens expressed on the endothelial cell surface are pivotal for autoimmune reactions, methods that detect antibodies only to endothelial cell surface molecules are required. Therefore, we developed a solubilized cell surface protein capture enzyme-linked immunosorbent assay (CSP-ELISA) that is able to detect antibodies against membrane proteins [18]. In this study, we evaluated the role of IgG and IgA isotypes of AECA to HUVEC and human glomerular endothelial cells (HGEC) in the diagnosis of SLE, and their association with clinical features and disease activity, using CSP-ELISA.

## Materials and Methods

### Patients

The study enrolled 76 SLE patients (65 women, 12 men) who were diagnosed according to the American College of Rheumatology criteria. Of the 76 SLE patients, 51 had biopsy-proven LN classified according to the International Society of Nephrology/Renal Pathology Society (ISN/RPS) LN classification [4]. Of the 76 SLE patients, 25 had no evidence of renal disease. Eighty healthy donors (healthy control; HC) and 42 patients with other forms of kidney disease with no immunoglobulin deposition evident with immunofluorescence (disease control; DC), including 32 patients with anti-endothelial cell antibodies (ANCA)-associated systemic vasculitis (AAV), 3 patients with minimal-change nephrotic syndrome, 2 patients with thin basement membrane disease, 4 patients with hypertension-related renal disease, and 1 patient with diabetic nephropathy, were enrolled as controls. Serum samples were obtained from the study participants with a written informed consent. This study was approved by the Ethics Committee of Fujita Health University (reference number HM16-052).

### Evaluation of disease activities

For each SLE patient, disease activity at the time of blood collection was assessed by using the Systemic Lupus Erythematosus Disease Activity Index (SLEDAI) score [19]. The histological lesion activity of LN patients was evaluated according to the ISN/RPS classification [4] by two independent nephrologists. Each individual component of the activity index, glomerular cellular proliferation, leukocyte infiltration, fibrinoid necrosis or karyorrhexis, and hyaline thrombi and wire loops, was scored on a scale of 0 to 3 according to the National Institutes of Health (NIH) scoring system [20]. All biopsy specimens contained at least 10 glomeruli.

### Cell culture and biotin labeling of cell surface proteins

Cell surface proteins were isolated from HUVEC (Lonza, Basel, Switzerland) and HGEC (DS Pharma Biomedical, Osaka, Japan) and were biotinylated as previously described [18]. Briefly, cultured cells were obtained by collagenase digestion. After cell surface proteins were biotin labeled, cells were homogenized using a Dounce homogenizer and solubilized with detergent buffer consisting of PBS containing 1% *n*-dodecyl-*β*-D maltopyranoside (DDM) (Dojindo, Kumamoto, Japan). Protein concentration was measured by Micro BCA™ protein assay kit (Thermoscientific, Waltham, MA).

### Immunofluorescence staining of HGEC

HGEC (5 × 10^5^) were incubated with blocking buffer (PBS with 2% normal goat serum, 0.2% BSA, and 0.02% NaN_3_) for 30 min at 4°C. Cells were incubated with serum (1:200 in PBS with 0.2% BSA and 0.02% NaN_3_) of LN patients with high and low serum titers of AECA for 1 h at 4°C. The cells were washed and Alexa488 conjugated anti-human IgG was added for a further 1 h. After washing two times, cells were fixed by Optilyse B (Beckman Coulter, Brea, CA) and analyzed using a flow cytometer (Beckman Coulter, Brea, CA) and fluorescence microscope. AECA to glomerular endothelial cells were detected by immunofluorescence on normal renal tissue cryosections (OriGene, Rockville, MD, USA) as described [21]. Briefly, 3μm cryosections were blocked with 10% goat serum for 30 min subsequently incubated with patient serum for 1 h. IgG and IgA binding to renal tissue were detected by using prediluted fluorescein isothiocyanate (FITC)-labeled polyclonal goat anti-human IgG or IgA (MBL, Nagoya, Japan). Sections were mounted using ProLongGold mounting medium (Invitrogen, Darmstadt, Germany) containing 4,6-diamidino-2-phenylindole (DAPI; 1:1000).

### Proliferation rate of HGEC

HGEC were incubated with serum of LN patients (IgG- or IgA-AECA positive) for 24 h at 37°C. The final serum concentration was 5%. After incubation, cell proliferation rates were analyzed using an IncuCyte ZOOM (Essen Bioscience, Ann Arbor, MI, USA). Proliferation rates were measured by confluence assay. Briefly, cell images were obtained at 0 and 24 h. The mean confluence rates were calculated from these cell images by using the IncuCyte ZOOM software. Proliferation rates were calculated per the following formula: Proliferation rates (%) = (after 24 h confluence rates/0 h confluence rates) × 100.

### AECA measurement using CSP-ELISA

IgG- and IgA-AECA titers were measured using CSP-ELISA with HUVEC and HGEC according to our previous study [18]. CSP-ELISA was performed using MaxiSorp immunomodules (Thermo Fisher Scientific, Rochester, NY, USA). Briefly, 100 μL DDM-solubilized protein solutions of 3.7 μg/mL HUVEC and 18 μg/mL HGEC were applied in 96-well micro-titer plates coated in 5 μg/mL NeutrAvidin^TM^ (Thermoscientific, Waltham, MA, USA) and incubated overnight at 4°C. After washing with PBS containing 0.01% DDM, 100 μL serum sample, diluted at 1:200 (IgG-HUVEC and IgG-HGEC), 1:100 (IgA-HUVEC), and 1:50 (IgA-HGEC) with PBS containing 0.5% skim milk and 0.01% DDM, was applied to the wells and incubated for 2 h at 4°C. The wells were washed again with 0.01% DDM in PBS and incubated with 100 μL of 1:5000 diluted HRP-conjugated anti-human IgG or IgA goat antibody (MBL, Nagoya, Japan). The substrate *o*-phenylenediamine dihydrochloride (Wako, Osaka, Japan) was added. After 20 min for IgG or 30 min for IgA, the optical density (OD) was read at 492 nm in an ELISA plate reader (Molecular Devices, Sunnyvale, CA, USA). All assays were performed in duplicate. Serum samples, showing the highest OD values of IgG-HUVEC, IgA-HUVEC, IgG-HGEC, and IgA-HGEC, were used as a standard reference serum to determine the standard curve (S1 Fig). All OD values were converted into arbitrary units (AU) by extrapolation from a standard curve with a local standard designated as 1 AU at 1:200 dilution in IgG-HUVEC and IgG-HGEC, and 1:100 dilution in IgA-HUVEC and IgA-HGEC.

### Cut-off points

A total of 112 healthy individuals were included to determine optimal cut-off values for AECA positivity. We selected the mean + 2 standard deviations (SD) of healthy controls as the cut-off AU values of AECA.

### Statistics

Statistical analysis was performed using SAS 6.10 (SAS Institute, Cary, NC, USA) and SPSS Statistics (IBM) software. Variables with a normal distribution are expressed as mean values ± SD, and asymmetrically distributed data are given as medians and interquartile range (IQR). We used *t* test or Mann–Whitney test for comparisons between two groups. Analysis of variance (ANOVA) or a Kruskal–Wallis test was used for comparison of quantitative data. Mann–Whitney test for nonparametric multiple comparison and Bonferroni correction were conducted. To define the clinical features correlated with IgA-AECA positivity in LN patients, multivariate logistic regression analysis was also performed. Receiver operating characteristic (ROC) curve analysis was used for calculating the area under the curve (AUC), sensitivity, and specificity. Unless otherwise specified, *P* values of less than 0.05 were considered statistically significant.

## Results

### Serum levels of IgG- and IgA-AECA detected by CSP- ELISA

**Fig 1:**
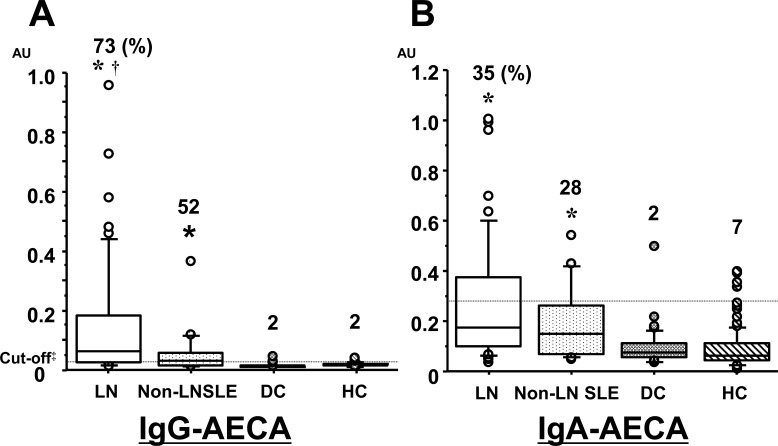
**Box plots of serum levels of IgG- (A) and IgA- (B) AECA detected by CSP-ELISA using HUVEC.** Titers of IgG- and IgA- AECA for HUVEC were significantly higher in LN and non-LN SLE patients than in the DC or HC groups. Percentage of positive ratio is shown above each box plot. *vs. DC or HC: *P* < 0.001, ^†^*vs*. non-LN SLE: *P* = 0.026. ^‡^Optimum cut-off points for predicting SLE were determined by mean + 2 SD for IgG-AECA and IgA-AECA levels. IgG-AECA serum level of 0.029 AU had a sensitivity of 0.66 and specificity of 0.99, and IgA-AECA serum level of 0.252 AU had a sensitivity of 0.33 and specificity of 0.90, for SLE prediction. IgG-AECA, immunoglobulin G anti-endothelial cell antibodies; IgA-AECA, immunoglobulin A anti-endothelial cell antibodies; HUVEC, human umbilical vein endothelial cells; LN, lupus nephritis; SLE, systemic lupus erythematosus; DC, disease control; HC, healthy control; AU, arbitrary unit.

The clinical characteristics of 51 LN patients and 25 SLE patients without nephritis (non-LN SLE) are presented in Table 1. Compared with non-LN SLE, LN patients had higher serum level of anti-double-stranded (ds) DNA antibodies (*P* = 0.015) and lower serum level of complement C3 (*P* < 0.001), reflecting higher disease activity (SLEDAI, *P* < 0.001). CSP-ELISA was performed to detect IgG- or IgA-AECA targeting HUVEC. All OD 492 values were converted into AU by extrapolation from a standard curve shown in S1 Fig. Sera were considered positive for IgG- and IgA-AECA to HUVEC if the AU value was higher than 0.0299 and 0.252, respectively. IgG-AECA prevalence in LN and non-LN SLE was 73% and 52%, respectively, compared with 2% in DC and 2% in HC (Fig 1A). IgA-AECA prevalence in LN and non-LN SLE was 35% and 28%, respectively, compared with 2% in DC and 7% in HC (Fig 1B). According to these cut-off values, IgG-AECA had a sensitivity of 0.66 and specificity of 0.99 and IgA-AECA a sensitivity of 0.33and specificity of 0.90 for differentiating SLE from HC subjects. IgG- and IgA-AECA titers for HUVEC were significantly higher in LN and non-LN SLE patients than those in the DC or HC (*P* <0.001) groups.

**Table 1:** Clinical characteristics of LN and SLE without nephritis.

	Lupus nephritis	SLE without nephritis	*P* value
No. of subjects	51	25	
Gender (M/F)	8/44	4/21	0.521
Age	36 (29–44)	42 (36–52)	0.037
sCr mg/dL	0.70 (0.58–0.83)	0.56 (0.52–0.60)	0.003
eGFR mL/min/1.73m^2^	83.8 **±** 28.0	96.4 **±** 26.4	0.068
Alb g/dL	3.1 **±** 0.7	4.0 **±** 0.6	<0.001
IgG mg/dL	1517 (942–1945)	1962 (1582–2154)	0.005
IgA mg/dL	265.2 **±** 103.0	316.1 **±** 151.2	0.110
C3 mg/dL	52.8 **±** 24.5	80.0 **±** 28.0	<0.001
dsDNA Ab IU/mL	35.0 (8.0–95.5)	7.5 (0–43.8)	0.015
UP (0–3+)	3 (2–3)	0	<0.001
UP g/gCr	2.7 (1.1–4.3)	0.02 (0.01–0.04)	<0.001
UOB (0–3+)	2 (0.5–3)	0	<0.001
URBC (sediments)	3 (1–15)	0 (0–1)	<0.001
SLEDAI	16 (12–22)	3 (2–4)	<0.001
Steroid (PSL mg)	10.0 (5–20)	5.3 (2.3–7.1)	0.039

sCr, serum creatinine; eGFR, estimated glomerular filtration rate; Alb, albumin; C3, complement component 3; Ab, antibody; UP, urine protein; UOB, urine occult blood; URBC, urine red blood cells; PSL, prednisolone

### AECA for glomerular endothelial cells

**Fig 2:**
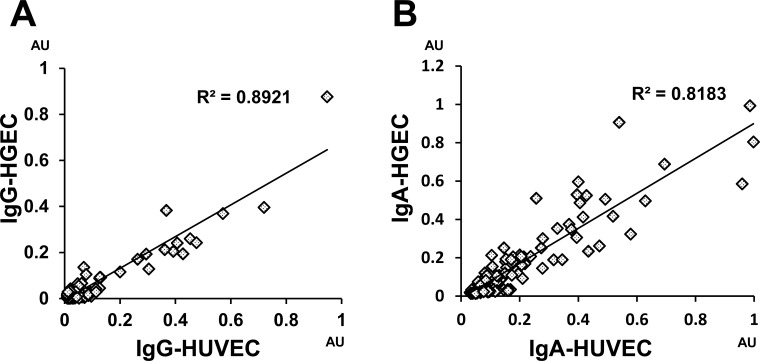
The association of AECA titers between HUVEC and HGEC. Significant correlation between AECA titers for HGEC and HUVEC (R^2^ = 0.89 for IgG-AECA and 0.82 for IgA-AECA) indicated AECA in LN patients recognize membrane proteins expressed on HGEC and HUVEC. HGEC, human glomerular endothelial cells

The AECA titers for HUVEC corresponded well with those for HGEC (R^2^ = 0.89 and 0.82 for IgG-AECA and IgA-AECA, respectively; Fig 2). The IgG- and IgA-AECA levels were not correlated well (ρ = 0.4936 and 0.2544 for HUVEC and HGEC, respectively; S2 Fig). To confirm whether IgG- and IgA-AECA recognize membrane proteins on HGEC, HGEC cells were incubated with serum of LN patients with high serum titer of IgG- or IgA-AECA and LN patients with low serum titer of IgG- or IgA-AECA. Positive staining for IgG or IgA from LN patients with high IgG- or IgA-AECA titer indicated that IgG- and IgA-AECA were strongly bound to membrane proteins on HGEC (S3A and S3B Fig). Furthermore, IgG and IgA binding to in situ human glomerular endothelial cells was not detected from a patient with LN with low serum titer of IgG- and IgA-AECA, but from a patient with high serum titer of IgG- and IgA-AECA (S3C Fig). HGEC were incubated with serum of patients with LN who are IgG- or IgA-AECA positive to detect functional activity of IgG- and IgA-AECA on HGEC. The proliferation of HGEC incubated with serum containing high titers of IgG or IgA-AECA was significantly decreased than those without AECA (S3D Fig). These results indicated that IgA-AECA recognize glomerular endothelial cells and may cause damage on the cells independently.

### Clinical significance of serum level of AECA in LN

**Fig 3:**
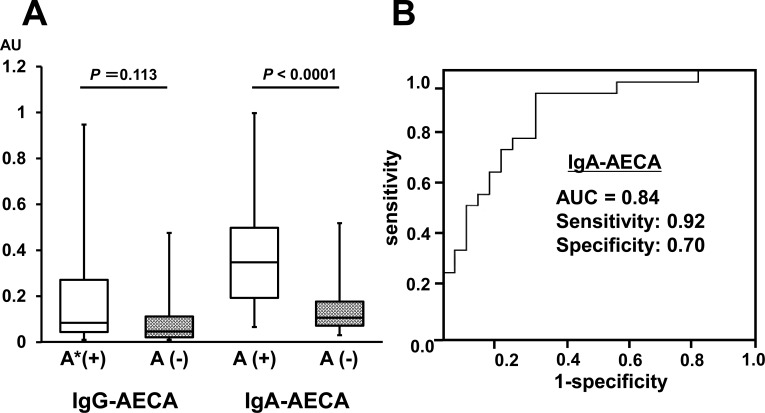
Correlation between AECA titer and histological evidence of active lesions defined by ISN/RPS classification in LN patients. A. The level of IgG-AECA did not correlate with active lesions, but the level of IgA-AECA for HUVEC did correlate with histological evidence of active lesions in LN patients (*P* < 0.001). *A: active lesion defined by ISN/RPS 2003 classification in LN. B. ROC curve analysis of IgA-AECA to differentiate LN patients with histological evidence of active lesions from those without. AUC was 0.84. The sensitivity as a diagnostic test for histological evidence of active lesions in LN patients was 0.92, with specificity of 0.70. AUC, area under curve.

To assess whether IgA-AECA are related to clinical features of LN, 33 LN patients without IgA-AECA and 18 LN patients with IgA-AECA were compared (Table 2). Anti-dsDNA antibody and IgG level were higher (*P* < 0.001, *P* = 0.002, respectively) and complement C3 level (*P* = 0.002) were lower in LN patients with than those without IgA-AECA. Multivariate logistic regression analysis showed that only the serum level of anti-dsDNA antibody is independently associated with patients with IgA-AECA (*P* = 0.016). IgA-AECA were more often detected in LN patients with than those without active glomerular lesions (78% vs. 30%, *P* = 0.001). Correlation of IgG- and IgA-AECA titers with histological evidence of active lesions is presented in Fig 3A. Although IgG-AECA titer did not correlate with active lesions (*P* = 0.11), the IgA-AECA titer correlated with histological evidence of active lesions in LN patients (*P* < 0.001). The ROC curves showed that the AUC value for IgA-AECA for distinguishing patients with active lesions from those without was 0.84 (Fig 3B). The sensitivity of IgA-AECA as a diagnostic test for active lesions in LN patients was 0.92, with a specificity of 0.70. To assess whether serum AECA level is influenced by immunosuppression therapy, the sera of four LN patients were collected after immunosuppression therapy. Of 4 patients with LN who had high serum levels of IgG- and IgA-AECA, three had decreased IgG- and IgA-AECA levels after immunosuppression therapy.

**Table 2:** Clinical characteristics of LN with IgA-AECA and without IgA-AECA.

	IgA-AECA negative	IgA-AECA positive	*P* value
No. of subjects	33	18	
Gender (M/F)	6/27	5/13	0.210
Age	36 (31–45)	34 (28–42)	0.244
sCr mg/dL	0.70 (0.53–0.81)	0.75 (0.63–0.93)	0.278
eGFR mL/min/1.73m^2^	86.8 **±** 28.5	78.3 **±** 27.1	0.309
Alb g/dL	3.1 **±** 0.7	2.9 **±** 0.6	0.399
IgG mg/dL	1068 (895–1732)	1799 (1558–2349)	0.002
IgA mg/dL	253.6 ± 94.0	285.9 **±** 117.5	0.256
C3 mg/dL	60.6 **±** 24.2	38.8 **±** 18.3	0.002
dsDNA Ab IU/mL	19.0 (8.0–51.7)	120.0 (54.5–300.0)	<0.001
UP (0–3+)	3 (2–3)	3 (1–3)	0.671
UP g/gCr	2.7 (1.2–4.2)	2.8 (0.7–4.5)	0.716
UOB (0–3+)	1 (0–2)	2.5 (1–3)	0.004
URBC (sediments)	3 (1–6)	11 (2–33)	0.081
SLEDAI	16 (12–20)	21 (15–25)	0.049
Steroid (PSL mg)	10.0 (6–20)	10.5 (0–20)	0.838
Histological active lesion (+\-)^a^	10/23 (30%)	14/4 (78%)	0.001
IgG deposition (+/-)^b^	25/3 (89%)	15/2 (88%)	0.382
IgA deposition (+/-)^b^	24/4 (86%)	17/0 (100%)	0.377
C3 deposition (+/-)^b^	27/1 (96%)	17/0 (100%)	0.137

^a^Histological evidence of active lesions defined by the ISN/RPS classification in LN patients: endocapillary hypercellularity with or without leukocyte infiltration and with substantial luminal reduction; karyorrhexis; fibrinoid necrosis; rupture of glomerular basement membrane; crescents, cellular or fibrocellular; subendothelial deposits identifiable by light microscopy (wireloops); intraluminal immune aggregates (hyaline thrombi)

^b^Judged by immunofluorescence microscopy

### Correlation between serum level of IgA-AECA and individual components of histological lesion activity

**Fig 4:**
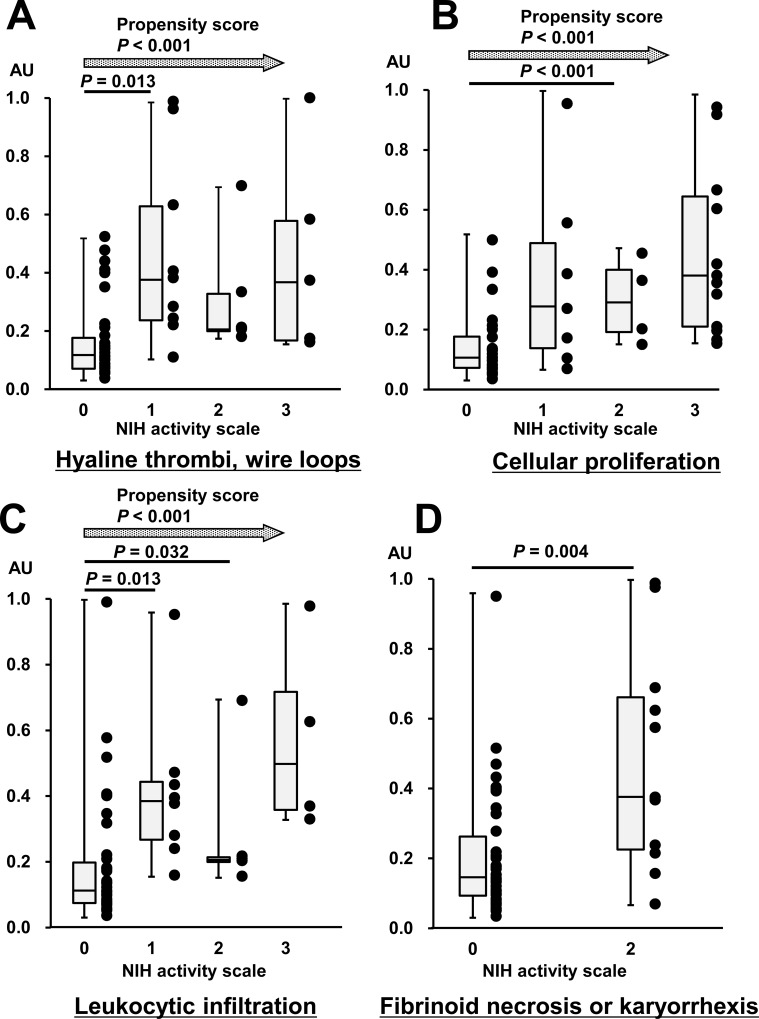
The association between serum level of IgA-AECA and each component of the activity index. A. Hyaline thrombi and wire loops. B. Cellular proliferation. C. Leukocytic infiltration. D. Fibrinoid necrosis or karyorrhexis. Higher serum level of IgA-AECA exhibits an increased propensity to higher scores of hyaline thrombi and wire loops, cellular proliferation, and leukocytic infiltration (*P* < 0.001).

To assess the relationship between IgA-AECA and individual components of active lesions, each component of active lesions was scored according to the NIH scoring system [20] and compared with the IgA-AECA titer (Fig 4). IgA-AECA levels were significantly higher in patients with score 1 than score 0 for hyaline thrombi and wire loops (*P* = 0.013), in patients with score 0 than score 2 for cellular proliferation (*P* < 0.001), in patients with score 1 than score 0 for leukocytic infiltration (*P* = 0.048), and in patients with fibrinoid necrosis or karyorrhexis than in those without lesions (*P* = 0.004). Higher serum level of IgA-AECA exhibited an increased propensity to higher scores for hyaline thrombi and wire loops, cellular proliferation, and leukocytic infiltration (*P* < 0.001).

## Discussion

Current laboratory markers of active renal disease include serum levels of anti-dsDNA antibodies, anti-C1q antibodies, and complement. These are not sensitive or specific enough for differentiating renal activity in LN [6, 16]. Therefore, novel biomarkers are necessary to enhance the diagnostic accuracy and sensitivity of disease activity in LN. In the present study, more than 70% of SLE patients had IgG-AECA detected by CSP-ELISA and the titer of IgA-AECA paralleled the disease activity of LN. IgG-AECA had a sensitivity of 0.92 and specificity of 0.85 for differentiating SLE from HC subjects. The sensitivity of IgA-AECA as a diagnostic test for active lesions in LN patients was 0.92, with a specificity of 0.70. Although these results need to be validated in large cohorts of patients with SLE and LN, detections of IgG- and IgA-AECA using CSP-ELISA are potentially sensitive and specific enough for detecting SLE and ongoing disease activity in LN.

More than 100 different antigens can be targeted in SLE. Although not all antibodies in SLE are pathogenic, variability in these antibodies influences the nature and severity of organ involvement in SLE [22]. The histological changes in LN are highly diverse, ranging from mild mesangial involvement, through proliferative forms of glomerulonephritis, to the membranous form. While the exact etiology triggering the different variants of LN remains unclear, antibodies to endothelial cells may play an important role because endothelial damage occurs as a central part of the pathology of LN. We identified that the level of IgG-AECA did not correlate with active disease, but the level of IgA-AECA did correlate with histological evidence of active lesions in LN patients. These results are in agreement with previous reports that evaluated IgG- and IgA-dsDNA antibodies in LN. It is generally believed that IgG anti-dsDNA antibodies play an important role in the pathogenesis of SLE, particularly in induction of LN [16, 23]. However, the association of IgG anti-dsDNA with kidney involvement or disease activity remains controversial [24, 25]. IgA isotype can enhance renal injury [16, 17, 26]. Interestingly, the prevalence of IgA anti-dsDNA reported in previous study [15, 17] is similar to the prevalence of IgA-AECA in SLE patients in the current study. Notably, our study included 32 patients with AAV who had severe endothelial damages without immunoglobulin deposition as disease control. Low serum levels of AECA in patient with AAV indicate that AECA is not formed secondary to vascular damage but is present specifically and relates with endothelial damage in patients with LN. Although IgA antibodies are unable to activate the classical complement pathway, recent insights suggest antibodies and IgA particularly may be implicated in the activation of the alternative and lectinic complement pathway in lupus glomeruli [27]. IgA autoantibodies may elicit non-classical complement activation and eventually contribute to LN aggravation [28].

A number of mechanisms have been proposed in which AECA may bind to endothelial cells and induce endothelial damage by complement activation, upregulation of adhesion molecules expression and cytokines, promotion of leukocyte adhesion, and induction of apoptosis and thrombosis [29–31]. In fact, patients with SLE display endothelial dysfunction and elevated levels of circulating apoptotic endothelial cells [32]. Endothelial damage is considered the first step in the pathogenesis of atherosclerosis, and atherosclerosis progresses more rapidly in SLE than in the general population [33, 34]. The high prevalence of IgG-AECA may be associated with endothelial damage and resultant atherosclerosis in patients with SLE. Further studies focused on the relationship between IgG-AECA and systemic vascular inflammation will be needed.

Further studies in large cohorts of SLE patients are also needed to evaluate the association between IgA-AECA and other clinical manifestations such as skin, central nervous system, and serosa involvement; vasculitis; thromboembolism; and hematological abnormalities. The serum levels of both IgG- and IgA-AECA decreased after immunosuppression therapy in a pilot analysis of four patients with LN, indicating that AECA may be a marker for therapeutic response in LN. Furthermore, IgA-AECA may be a target for therapeutic intervention. Further studies evaluating the significance of AECA on therapeutic intervention should be performed. The main limitation of our study is we did not identify antigens for AECA on endothelial cells. The characterization of candidate antigens for AECA is complicated because most membranous proteins form multiprotein complexes. Although target antigens were not characterized, the correlation between AECA titers and disease activity suggests AECA to be an important clinical marker. In conclusion, our study demonstrated high sensitivity and selectivity of IgG-AECA in the diagnosis of SLE, and of IgA-AECA in the diagnosis of histological evidence of active lesions in LN. The aforementioned data suggest that IgA-AECA directly recognize the membrane protein of endothelial cells and are associated with endothelial damage in LN.

## Supporting Information

S1 Dataset Data set used for Figs 1–4, S1 and S4 Figs, and Table 2. (PDF)

S1 Fig Standard curves of CSP-ELISA. Standard curves of CSP-ELISA for IgG-HUVEC, IgA-HUVEC, IgG-HGEC, and IgA-HGEC were generated from serum showing the highest OD 492 nm values. Second-order polynomial trend lines were produced best fit with the data.
(TIF)

S2 Fig The association of IgG and IgA-AECA. A. The level of IgG- and IgA-HUVEC is not correlated well. B. The level of IgG- and IgA-HGEC is not correlated well.
(TIF)

S3 Fig AECA from patients with LN recognized membrane proteins on HGEC. A. Immunocytochemical analysis showed positive staining in HGEC for IgG using serum from LN patients with high titer of AECA. B. Flow cytometric analysis using serum of LN patients demonstrated that IgG- or IgA-AECA bind strongly to HGEC. C. Serum IgG- and IgA-AECA to glomerular endothelial cells were detected via immunofluorescence on normal renal tissue cryosections. D. The proliferation of HGEC incubated with serum containing high titer of IgG or IgA-AECA were significantly decreased than those without AECA. *P < 0.05, **P < 0.01 vs IgA and IgG negative serum (Scheffe test).
(TIF)

S4 Fig Serum levels of IgG- and IgA- AECA in patients with LN before and after immunosuppression therapy. (TIF)
