# Supplementary material for: The Level of IgA Antibodies to Endothelial Cells Correlates with Histological Evidence of Disease Activity in Patients with Lupus Nephritis
Source: PLoS One. 2016 Oct 27;11(10):e0163085. doi: 10.1371/journal.pone.0163085 (PMC5082850; doi:10.1371/journal.pone.0163085)
Supplement: S1 Dataset — (PDF) [file pone.0163085.s001.pdf]

IgG-HUVEC					IGA-HUVEC				
	LUPUS	nonLN	DC	HC		Lupus	nonLN	DC	HC
	au	au	au	au		au	au	au	au
	0.01151	0.01364	0.01269	0.02491		0.07348	0.4159	0.12128	0.02713
	0.45315	0.01063	0.01557	0.02435		0.31515	0.12131	0.05907	0.0148
	0.06914	0.01533	0.01471	0.026		0.07094	0.27292	0.06365	0.02658
	0.07752	0.05219	0.01	0.01648		0.39367	0.18661	0.08737	0.04578
	0.12633	0.02058	0.02459	0.01955		0.57821	0.53876	0.49157	0.02051
	0.57111	0.00989	0.01269	0.02669		0.99734	0.15259	0.03961	0.08278
	0.42717	0.07497	0.0154	0.02738		0.34473	0.05294	0.05753	0.02187
	0.04691	0.08577	0.01153	0.01997		0.10354	0.42757	0.08385	0.01936
	0.12998	0.05083	0.02062	0.02778		0.36774	0.14579	0.03522	0.04598
	0.09624	0.03698	0.0133	0.02918		0.21728	0.05536	0.06473	0.00729
	0.02143	0.01013	0.01212	0.02029		0.07767	0.14865	0.0983	0.15708
	0.0213	0.08522	0.01087	0.02131		0.04856	0.07985	0.05472	0.02288
	0.05793	0.0499	0.01178	0.02743		0.09581	0.27832	0.14531	0.05234
	0.30367	0.11311	0.00987	0.02549		0.06308	0.18773	0.17245	0.04451
	0.08496	0.11326	0.01425	0.01196		0.1177	0.06191	0.0911	0.0681
	0.00997	0.04052	0.0099	0.01867		0.10273	0.25574	0.05396	0.04473
	0.40655	0.01408	0.01351	0.01776		0.1068	0.17004	0.12259	0.0367
	0.367	0.01665	0.01072	0.01832		0.1675	0.0871	0.16805	0.0378
	0.06406	0.01523	0.01177	0.01946		0.20575	0.0715	0.11001	0.00695
	0.20017	0.36124	0.02094	0.02027		0.51811	0.12732	0.04699	0.05413
	0.09365	0.01412	0.01015	0.02192		0.1994	0.04549	0.0487	0.14285
	0.01006	0.01605	0.01337	0.01438		0.09271	0.06368	0.08713	0.09175
	0.06183	0.03643	0.01052	0.01995		0.47168	0.39484	0.03228	0.05837
	0.04477	0.01561	0.01048	0.01867		0.15111	0.05459	0.05421	0.10829
	0.47578	0.03097	0.01102	0.01603		0.06727	0.19421	0.11606	0.05208
	0.04725		0.01006	0.02152		0.39948		0.0964	0.38981
	0.06296		0.01095	0.01662		0.13288		0.09735	0.02742
	0.12609		0.01519	0.02069		0.43405		0.09609	0.03986
	0.02262		0.01069	0.01991		0.21368		0.04699	0.06183
	0.04217		0.01694	0.01272		0.15454		0.20941	0.03352
	0.26337		0.01205	0.01362		0.95884		0.0492	0.05897
	0.09157		0.01017	0.01518		0.17346		0.06555	0.0069
	0.01091		0.01035	0.0231		0.03015		0.09234	0.0476
	0.02773		0.00996	0.02749		0.27728		0.03715	0.01507
	0.01867		0.01617	0.01854		0.04627		0.15902	0.05902
	0.05152		0.00986	0.02343		0.17702		0.06365	0.39507
KD130	0.94751		0.01185	0.02479		0.98503		0.05447	0.11315
	0.03907		0.04319	0.03019		0.40486		0.04105	0.04755
	0.03336		0.0118	0.02205		0.08074		0.06912	0.0333
	0.01853		0.01059	0.01751		0.05289		0.0492	0.06013
	0.03423		0.0099	0.02521		0.17686		0.16018	0.10432
	0.01605		0.01233	0.01707		0.23676		0.07619	0.09079
	0.07179			0.02464		0.06598			0.05787
	0.39229			0.02195		0.69385			0.05021
	0.12785			0.02018		0.13686			0.06384
	0.02152			0.02768		0.32766			0.2415
	0.29453			0.026		0.62817			0.14204
	0.71994			0.02361		0.37589			0.35197
	0.02162			0.02418		0.20479			0.06274
	0.01725			0.02185		0.12966			0.05342
	0.03685			0.02461		0.1413			0.04149
				0.01008					0.06541
				0.0233					0.04107
				0.02522					0.16135
				0.0274					0.03722
				0.01961					0.05226
				0.02076					0.06247
				0.01072					0.16433
				0.02131					0.17625
				0.02566					0.06804
				0.0241					0.09055
				0.01893					0.07732
				0.01885					0.17145
				0.01038					0.1039
				0.02264					0.10553
				0.02206					0.12024
				0.02608					0.08118
				0.02167					0.02084
				0.02534					0.07263
				0.01326					0.07376
				0.0178					0.04382
				0.01492					0.0069
				0.0195					0.01339
				0.02419					0.26977
				0.01515					0.14673
				0.02329					0.17447
				0.01553					0.04313
				0.01872					0.04758
				0.02479					0.17633
				0.02126					0.02454
				0.01241					0.06381
				0.02323					0.05383
				0.01814					0.06119
				0.02399					0.06063
				0.02016					0.08254
				0.02383					0.07359
				0.01878					0.08493
				0.01777					0.04316
				0.01028					0.0207
				0.0208					0.04868
				0.01056					0.06642
				0.01417					0.02897
				0.01169					0.07217
				0.01105					0.05631
				0.01309					0.04081
				0.01458					0.08773
				0.01172					0.0292
				0.01764					0.38528
				0.01464					0.17269
				0.01357					0.0849
				0.01198					0.14419
				0.00996					0.12549
				0.0149					0.09581
				0.01901					0.13778
				0.00986					0.04627
				0.03407					0.02821
				0.01761					0.05655
				0.0145					0.03569
				0.0128					0.26648
				0.01412					0.04974
				0.01902					0.0538
				0.01526					0.11268
				0.01643					0.1739
				0.01165					0.18903
				0.01508					0.32879
				0.01256					0.26732
				0.01074					0.25407
				0.01057					0.0903
				0.01639					0.08777
				0.01702					0.21262
				0.01139					0.12173
				0.01169					0.0069
									0.03343
									0.03788
									0.0721
									0.11805
									0.07469
									0.11584
									0.05668

疾患1：SL	huvecigg	au	hugecigg	au	huveciga	au	hugeciga	au
1	0.1875	0.01151	0.1489	0.01842	0.14925	0.07348	0.41065	0.07457
1	1.39335	0.45315	0.9098	0.26032	0.447	0.31515	0.7347	0.1903
1	0.67245	0.06914	0.7398	0.13625	0.14475	0.07094	0.29755	0.04756
1	0.70085	0.07752	0.68605	0.10541	0.51755	0.39367	0.96425	0.3067
1	0.8394	0.12633	0.65315	0.08852	0.66085	0.57821	0.99405	0.32391
1	1.53565	0.57111	1.02765	0.36996	0.92005	0.99734	1.6291	0.80486
1	1.35955	0.42717	0.82705	0.1949	0.47445	0.34473	0.7349	0.19039
1	0.5854	0.04691	0.6023	0.06538	0.19865	0.10354	0.78545	0.21358
1	0.84845	0.12998	0.66005	0.09194	0.49505	0.36774	1.0788	0.37546
1	0.75855	0.09624	0.4094	0.01038	0.3464	0.21728	0.6855	0.16907
1	0.4405	0.02143	0.469	0.02183	0.15655	0.07767	0.4046	0.07295
1	0.43945	0.0213	0.2449	0.00445	0.1022	0.04856	0.24765	0.03785
1	0.6311	0.05793	0.4316	0.01406	0.18655	0.09581	0.4098	0.07434
1	1.1821	0.30367	0.7278	0.12902	0.13045	0.06308	0.3532	0.05998
1	0.72465	0.08496	0.4735	0.0229	0.2199	0.1177	0.37025	0.06412
1	0.27475	0.00997	0.24625	0.00434	0.1974	0.10273	0.4405	0.08285
1	1.33195	0.40655	0.8873	0.24158	0.20365	0.1068	0.45	0.08559
1	1.27695	0.367	1.04085	0.38345	0.2873	0.1675	0.61535	0.14106
1	0.65425	0.06406	0.53455	0.04015	0.3333	0.20575	0.67155	0.16329
1	1.0015	0.20017	0.70525	0.11597	0.61695	0.51811	1.14275	0.41694
1	0.75095	0.09365	0.48855	0.02667	0.32595	0.1994	0.78955	0.21552
1	0.2329	0.01006	0.23885	0.00495	0.1816	0.09271	0.34975	0.05916
1	0.646	0.06183	0.6058	0.06686	0.58135	0.47168	0.8831	0.26229
1	0.5758	0.04477	0.38755	0.00741	0.2662	0.15111	0.71075	0.1798
1	1.422	0.47578	0.8893	0.24322	0.13815	0.06727	0.31635	0.05157
1	0.5869	0.04725	0.42575	0.01303	0.5225	0.39948	1.388	0.59654
1	0.6502	0.06296	0.3896	0.00766	0.24155	0.13288	0.4492	0.08535
1	0.8388	0.12609	0.551	0.04569	0.55125	0.43405	0.82855	0.23444
1	0.44965	0.02262	0.3261	0.00266	0.34235	0.21368	0.768	0.20542
1	0.5637	0.04217	0.3979	0.00873	0.2707	0.15454	0.6374	0.14958
1	1.1163	0.26337	0.7936	0.17116	0.8988	0.95884	1.375	0.58621
1	0.43645	0.02093	0.5428	0.04288	0.2028	0.10625	0.65095	0.15494
1	0.7448	0.09157	0.4551	0.01872	0.29475	0.17346	0.6884	0.17029
1	0.2015	0.01091	0.1928	0.01043	0.06205	0.03015	0.13255	0.02059
1	0.48505	0.02773	0.32625	0.00266	0.41005	0.27728	0.6286	0.14615
1	0.41705	0.01867	0.3121	0.00231	0.0975	0.04627	0.2907	0.04615
1	0.60525	0.05152	0.5072	0.03178	0.29915	0.17702	0.71325	0.18089
1	1.90975	0.94751	1.4215	0.87687	0.9133	0.98503	1.8218	0.994
1	0.5486	0.03907	0.48245	0.0251	0.52705	0.40486	1.24405	0.48718
1	0.51855	0.03336	0.48835	0.02662	0.1618	0.08074	0.5628	0.12183
1	0.4158	0.01853	0.30705	0.00225	0.1109	0.05289	0.35445	0.06028
1	0.52335	0.03423	0.454	0.01848	0.29895	0.17686	0.76765	0.20526
1	0.39115	0.01605	0.30155	0.00223	0.36785	0.23676	0.77235	0.20744
1	0.68165	0.07179	0.3661	0.00516	0.1358	0.06598	0.3045	0.04902
1	1.31245	0.39229	0.84095	0.20522	0.73955	0.69385	1.49945	0.68893
1	0.8432	0.12785	0.66255	0.09319	0.24705	0.13686	0.46525	0.09008
1	0.4412	0.02152	0.37675	0.0062	0.45875	0.32766	1.0437	0.35364
1	1.1676	0.29453	0.82515	0.19351	0.6957	0.62817	1.25855	0.49768
1	1.69525	0.71994	1.05355	0.39666	0.5022	0.37589	1.03535	0.34855
1	0.442	0.02162	0.3279	0.00272	0.3322	0.20479	0.77275	0.20763
1	0.40355	0.01725	0.39255	0.00803	0.23705	0.12966	0.52585	0.1092
1	0.5373	0.03685	0.37015	0.00553	0.2531	0.1413	0.49395	0.09889
2	0.36175	0.01364	0.28135	0.00251	0.5363	0.4159	1.13705	0.41315
2	0.30415	0.01063	0.22545	0.00624	0.22515	0.12131	0.40555	0.0732
2	0.3831	0.01533	0.28095	0.00252	0.40565	0.27292	0.8644	0.25257
2	0.60805	0.05219	0.31195	0.00231	0.3108	0.18661	0.594	0.13307
2	0.4336	0.02058	0.4297	0.01372	0.6323	0.53876	1.73575	0.90705
2	0.24725	0.00989	0.2903	0.00231	0.26815	0.15259	0.74375	0.19435
2	0.6924	0.07497	0.39905	0.00889	0.111	0.05294	0.23085	0.03488
2	0.72715	0.08577	0.4314	0.01403	0.54595	0.42757	1.2967	0.52588
2	0.60235	0.05083	0.5787	0.05587	0.25915	0.14579	0.86455	0.25264
2	0.53795	0.03698	0.3272	0.0027	0.11575	0.05536	0.23185	0.03505
2	0.28495	0.01013	0.19545	0.01004	0.26295	0.14865	0.55965	0.12072
2	0.72545	0.08522	0.45165	0.01799	0.1603	0.07985	0.36545	0.06294
2	0.5984	0.0499	0.4447	0.01656	0.4111	0.27832	0.95335	0.30053
2	0.80535	0.11311	0.54085	0.04223	0.31215	0.18773	0.58935	0.13136
2	0.80575	0.11326	0.4981	0.02923	0.12825	0.06191	0.2702	0.04207
2	0.55575	0.04052	0.5017	0.03022	0.388	0.25574	1.2761	0.51056
2	0.3678	0.01408	0.2246	0.00633	0.2905	0.17004	0.73685	0.19126
2	0.3975	0.01665	0.2317	0.0056	0.17245	0.0871	0.4315	0.0803
2	0.382	0.01523	0.34625	0.00364	0.14575	0.0715	0.4477	0.08492
2	1.2687	0.36124	0.85105	0.21289	0.23375	0.12732	0.50585	0.10267
2	0.36825	0.01412	0.29675	0.00224	0.0959	0.04549	0.18755	0.02794
2	0.3912	0.01605	0.2828	0.00247	0.13155	0.06368	0.33615	0.056
2	0.5351	0.03643	0.37865	0.0064	0.51855	0.39484	1.30235	0.53012
2	0.38635	0.01561	0.3598	0.00461	0.11425	0.05459	0.2548	0.03916
2	0.50485	0.03097	0.3037	0.00223	0.31985	0.19421	0.5487	0.11692
3	0.1661	0.01269	0.18665	0.01139	0.2251	0.12128	0.51865	0.10682
3	0.3859	0.01557	0.2595	0.00345	0.1229	0.05907	0.29985	0.04804
3	0.13795	0.01471	0.1603	0.01608	0.1315	0.06365	0.39705	0.07095
3	0.2369	0.01	0.24645	0.00433	0.1729	0.08737	0.5254	0.10905
3	0.46405	0.02459	0.36795	0.00533	0.5968	0.49157	1.2697	0.50584
3	0.16595	0.01269	0.1969	0.00982	0.0834	0.03961	0.21855	0.03281
3	0.12975	0.0154	0.17895	0.01266	0.11995	0.05753	0.41545	0.07587
3	0.187	0.01153	0.22695	0.00608	0.16705	0.08385	0.4404	0.08282
3	0.07975	0.02062	0.08875	0.03371	0.0737	0.03522	0.22385	0.03369
3	0.1567	0.0133	0.18705	0.01133	0.1335	0.06473	0.3422	0.05739
3	0.338	0.01212	0.169	0.01443	0.1905	0.0983	0.1845	0.02749
3	0.311	0.01087	0.2465	0.00432	0.1145	0.05472	0.106	0.01762
3	0.182	0.01178	0.1395	0.02047	0.2585	0.14531	0.23	0.03474
3	0.252	0.00987	0.1625	0.01566	0.2935	0.17245	0.527	0.10958
3	0.37	0.01425	0.2015	0.00917	0.179	0.0911	0.144	0.02198
3	0.268	0.0099	0.229	0.00587	0.113	0.05396	0.108	0.01783
3	0.36	0.01351	0.2135	0.0076	0.227	0.12259	0.202	0.03014
3	0.307	0.01072	0.1845	0.01174	0.288	0.16805	0.2335	0.03534
3	0.3315	0.01177	0.203	0.00896	0.2085	0.11001	0.238	0.03613
3	0.4365	0.02094	0.3535	0.00413	0.099	0.04699	0.096	0.01661
3	0.2275	0.01015	0.1365	0.02116	0.1025	0.0487	0.1005	0.01706
3	0.358	0.01337	0.2645	0.00317	0.1725	0.08713	0.174	0.02598
3	0.213	0.01052	0.1505	0.01808	0.067	0.03228	0.078	0.01491
3	0.2995	0.01048	0.1645	0.01527	0.1135	0.05421	0.115	0.01859
3	0.315	0.01102	0.191	0.01071	0.2175	0.11606	0.1705	0.02549
3	0.281	0.01006	0.1825	0.01206	0.1875	0.0964	0.162	0.02432
3	0.2005	0.01095	0.1585	0.01644	0.189	0.09735	0.1875	0.02793
3	0.3815	0.01519	0.275	0.00271	0.187	0.09609	0.1995	0.02975
3	0.306	0.01069	0.24	0.00485	0.099	0.04699	0.089	0.01593
3	0.4005	0.01694	0.3585	0.00451	0.3375	0.20941	0.4735	0.09257
3	0.177	0.01205	0.1035	0.0295	0.1035	0.0492	0.076	0.01474
3	0.2265	0.01017	0.1495	0.01829	0.135	0.06555	0.1125	0.01831
3	0.2945	0.01035	0.143	0.01969	0.181	0.09234	0.1725	0.02577
3	0.2395	0.00996	0.1585	0.01644	0.078	0.03715	0.072	0.01439
3	0.3925	0.01617	0.242	0.00468	0.2765	0.15902	0.237	0.03595
3	0.26	0.00986	0.18	0.01248	0.1315	0.06365	0.1565	0.02359
3	0.333	0.01185	0.172	0.01388	0.114	0.05447	0.097	0.01671
3	0.5685	0.04319	0.3855	0.00717	0.0865	0.04105	0.0995	0.01696
3	0.332	0.0118	0.185	0.01165	0.1415	0.06912	0.1565	0.02359
3	0.303	0.01059	0.191	0.01071	0.1035	0.0492	0.0885	0.01588
3	0.2465	0.0099	0.153	0.01756	0.278	0.16018	0.1985	0.0296
3	0.172	0.01233	0.105	0.02908	0.154	0.07619	0.1575	0.02372

igg activ€	au	igg activ€	au	iga activ€	au	iga activ€	au
0.70085	0.07752	0.1875	0.01151	0.51755	0.39367	0.14925	0.07348
0.8394	0.12633	1.39335	0.45315	0.66085	0.57821	0.447	0.31515
1.53565	0.57111	0.67245	0.06914	0.92005	0.99734	0.14475	0.07094
0.84845	0.12998	1.35955	0.42717	0.49505	0.36774	0.47445	0.34473
0.27475	0.00997	0.5854	0.04691	0.1974	0.10273	0.19865	0.10354
1.27695	0.367	0.75855	0.09624	0.2873	0.1675	0.3464	0.21728
0.65425	0.06406	0.4405	0.02143	0.3333	0.20575	0.15655	0.07767
0.75095	0.09365	0.43945	0.0213	0.32595	0.1994	0.1022	0.04856
0.646	0.06183	0.6311	0.05793	0.58135	0.47168	0.18655	0.09581
0.5758	0.04477	1.1821	0.30367	0.2662	0.15111	0.13045	0.06308
0.5869	0.04725	0.72465	0.08496	0.5225	0.39948	0.2199	0.1177
0.8388	0.12609	1.33195	0.40655	0.55125	0.43405	0.20365	0.1068
0.44965	0.02262	1.0015	0.20017	0.34235	0.21368	0.61695	0.51811
0.5637	0.04217	0.2329	0.01006	0.2707	0.15454	0.1816	0.09271
1.1163	0.26337	1.422	0.47578	0.8988	0.95884	0.13815	0.06727
0.7448	0.09157	0.6502	0.06296	0.29475	0.17346	0.24155	0.13288
0.48505	0.02773	0.2015	0.01091	0.41005	0.27728	0.06205	0.03015
1.90975	0.94751	0.41705	0.01867	0.9133	0.98503	0.0975	0.04627
0.39115	0.01605	0.60525	0.05152	0.36785	0.23676	0.29915	0.17702
0.68165	0.07179	0.5486	0.03907	0.1358	0.06598	0.52705	0.40486
1.31245	0.39229	0.51855	0.03336	0.73955	0.69385	0.1618	0.08074
0.4412	0.02152	0.4158	0.01853	0.45875	0.32766	0.1109	0.05289
1.1676	0.29453	0.52335	0.03423	0.6957	0.62817	0.29895	0.17686
1.69525	0.71994	0.8432	0.12785	0.5022	0.37589	0.24705	0.13686
		0.442	0.02162			0.3322	0.20479
		0.40355	0.01725			0.23705	0.12966
		0.5373	0.03685			0.2531	0.1413

active	**huvec -ig**	au	**hgec -iga**	au
1	0.51755	0.39367	0.96425	0.3067
1	0.66085	0.57821	0.99405	0.32391
1	0.92005	0.99734	1.6291	0.80486
1	0.49505	0.36774	1.0788	0.37546
1	0.1974	0.10273	0.4405	0.08285
1	0.2873	0.1675	0.61535	0.14106
1	0.3333	0.20575	0.67155	0.16329
1	0.32595	0.1994	0.78955	0.21552
1	0.58135	0.47168	0.8831	0.26229
1	0.2662	0.15111	0.71075	0.1798
1	0.5225	0.39948	1.388	0.59654
1	0.55125	0.43405	0.82855	0.23444
1	0.34235	0.21368	0.768	0.20542
1	0.2707	0.15454	0.6374	0.14958
1	0.8988	0.95884	1.375	0.58621
1	0.29475	0.17346	0.6884	0.17029
1	0.41005	0.27728	0.6286	0.14615
1	0.9133	0.98503	1.8218	0.994
1	0.36785	0.23676	0.77235	0.20744
1	0.1358	0.06598	0.3045	0.04902
1	0.73955	0.69385	1.49945	0.68893
1	0.45875	0.32766	1.0437	0.35364
1	0.6957	0.62817	1.25855	0.49768
1	0.5022	0.37589	1.03535	0.34855
0	0.14925	0.07348	0.41065	0.07457
0	0.447	0.31515	0.7347	0.1903
0	0.14475	0.07094	0.29755	0.04756
0	0.47445	0.34473	0.7349	0.19039
0	0.19865	0.10354	0.78545	0.21358
0	0.3464	0.21728	0.6855	0.16907
0	0.15655	0.07767	0.4046	0.07295
0	0.1022	0.04856	0.24765	0.03785
0	0.18655	0.09581	0.4098	0.07434
0	0.13045	0.06308	0.3532	0.05998
0	0.2199	0.1177	0.37025	0.06412
0	0.20365	0.1068	0.45	0.08559
0	0.61695	0.51811	1.14275	0.41694
0	0.1816	0.09271	0.34975	0.05916
0	0.13815	0.06727	0.31635	0.05157
0	0.24155	0.13288	0.4492	0.08535
0	0.06205	0.03015	0.13255	0.02059
0	0.0975	0.04627	0.2907	0.04615
0	0.29915	0.17702	0.71325	0.18089
0	0.52705	0.40486	1.24405	0.48718
0	0.1618	0.08074	0.5628	0.12183
0	0.1109	0.05289	0.35445	0.06028
0	0.29895	0.17686	0.76765	0.20526
0	0.24705	0.13686	0.46525	0.09008
0	0.3322	0.20479	0.77275	0.20763
0	0.23705	0.12966	0.52585	0.1092
0	0.2531	0.1413	0.49395	0.09889

celluler	huveciga	au end	wire loop	huveciga	au wire	fibrinoid	huveciga	au necros	cressent	huveciga	au cresse	leukocyt	huveciga	au leuco
0	0.36785	0.23676	0	0.55125	0.43405	0	0.2873	0.1675	0	0.14475	0.07094	0	0.66085	0.57821
0	0.14475	0.07094	0	0.51755	0.39367	0	0.55125	0.43405	0	0.47445	0.34473	0	0.92005	0.99734
0	0.47445	0.34473	0	0.58135	0.47168	0	0.45875	0.32766	0	0.19865	0.10354	0	0.1974	0.10273
0	0.19865	0.10354	0	0.2662	0.15111	0	0.51755	0.39367	0	0.3464	0.21728	0	0.2873	0.1675
0	0.3464	0.21728	0	0.14475	0.07094	0	0.32595	0.1994	0	0.15655	0.07767	0	0.5225	0.39948
0	0.15655	0.07767	0	0.47445	0.34473	0	0.1974	0.10273	0	0.1022	0.04856	0	0.29475	0.17346
0	0.1022	0.04856	0	0.19865	0.10354	0	0.58135	0.47168	0	0.18655	0.09581	0	0.1358	0.06598
0	0.18655	0.09581	0	0.3464	0.21728	0	0.2662	0.15111	0	0.13045	0.06308	0	0.14925	0.07348
0	0.13045	0.06308	0	0.15655	0.07767	0	0.3333	0.20575	0	0.2199	0.1177	0	0.447	0.31515
0	0.2199	0.1177	0	0.1022	0.04856	0	0.8988	0.95884	0	0.20365	0.1068	0	0.14475	0.07094
0	0.20365	0.1068	0	0.18655	0.09581	0	0.29475	0.17346	0	0.61695	0.51811	0	0.47445	0.34473
0	0.61695	0.51811	0	0.13045	0.06308	0	0.41005	0.27728	0	0.1816	0.09271	0	0.19865	0.10354
0	0.1816	0.09271	0	0.2199	0.1177	0	0.5225	0.39948	0	0.13815	0.06727	0	0.3464	0.21728
0	0.13815	0.06727	0	0.20365	0.1068	0	0.14475	0.07094	0	0.24155	0.13288	0	0.15655	0.07767
0	0.24155	0.13288	0	0.61695	0.51811	0	0.47445	0.34473	0	0.06205	0.03015	0	0.1022	0.04856
0	0.2028	0.10625	0	0.1816	0.09271	0	0.19865	0.10354	0	0.0975	0.04627	0	0.18655	0.09581
0	0.06205	0.03015	0	0.13815	0.06727	0	0.3464	0.21728	0	0.29915	0.17702	0	0.13045	0.06308
0	0.0975	0.04627	0	0.24155	0.13288	0	0.15655	0.07767	0	0.52705	0.40486	0	0.2199	0.1177
0	0.29915	0.17702	0	0.06205	0.03015	0	0.1022	0.04856	0	0.1618	0.08074	0	0.20365	0.1068
0	0.52705	0.40486	0	0.0975	0.04627	0	0.18655	0.09581	0	0.1109	0.05289	0	0.61695	0.51811
0	0.1618	0.08074	0	0.29915	0.17702	0	0.13045	0.06308	0	0.29895	0.17686	0	0.1816	0.09271
0	0.1109	0.05289	0	0.52705	0.40486	0	0.2199	0.1177	0	0.24705	0.13686	0	0.13815	0.06727
0	0.29895	0.17686	0	0.1618	0.08074	0	0.20365	0.1068	0	0.3322	0.20479	0	0.24155	0.13288
0	0.24705	0.13686	0	0.1109	0.05289	0	0.61695	0.51811	0	0.23705	0.12966	0	0.2028	0.10625
0	0.3322	0.20479	0	0.29895	0.17686	0	0.1816	0.09271	0	0.2531	0.1413	0	0.06205	0.03015
0	0.23705	0.12966	0	0.24705	0.13686	0	0.13815	0.06727	0	0.8988	0.95884	0	0.0975	0.04627
1	0.92005	0.99734	0	0.3322	0.20479	0	0.24155	0.13288	0	0.41005	0.27728	0	0.29915	0.17702
1	0.66085	0.57821	0	0.23705	0.12966	0	0.06205	0.03015	0	0.5225	0.39948	0	0.52705	0.40486
1	0.1358	0.06598	0	0.2531	0.1413	0	0.0975	0.04627	0	0.32595	0.1994	0	0.1618	0.08074
1	0.1974	0.10273	0	0.1358	0.06598	0	0.29915	0.17702	0	0.3333	0.20575	0	0.1109	0.05289
1	0.29475	0.17346	1	0.1974	0.10273	0	0.52705	0.40486	0	0.29475	0.17346	0	0.29895	0.17686
1	0.41005	0.27728	1	0.8988	0.95884	0	0.1618	0.08074	0	0.73955	0.69385	0	0.24705	0.13686
1	0.5225	0.39948	1	0.41005	0.27728	0	0.1109	0.05289	0	0.49505	0.36774	0	0.3322	0.20479
2	0.5022	0.37589	1	0.5225	0.39948	0	0.29895	0.17686	0	0.92005	0.99734	0	0.23705	0.12966
2	0.58135	0.47168	1	0.9133	0.98503	0	0.24705	0.13686	0	0.66085	0.57821	1	0.51755	0.39367
2	0.2662	0.15111	1	0.6957	0.62817	0	0.3322	0.20479	2	0.51755	0.39367	1	0.58135	0.47168
2	0.3333	0.20575	1	0.34235	0.21368	0	0.23705	0.12966	2	0.58135	0.47168	1	0.55125	0.43405
3	0.9133	0.98503	1	0.5022	0.37589	0	0.2531	0.1413	2	0.2662	0.15111	1	0.2707	0.15454
3	0.6957	0.62817	1	0.36785	0.23676	2	0.9133	0.98503	2	0.1358	0.06598	1	0.8988	0.95884
3	0.2707	0.15454	2	0.45875	0.32766	2	0.6957	0.62817	2	0.1974	0.10273	1	0.41005	0.27728
3	0.2873	0.1675	2	0.32595	0.1994	2	0.2707	0.15454	2	0.34235	0.21368	1	0.36785	0.23676
3	0.34235	0.21368	2	0.3333	0.20575	2	0.34235	0.21368	2	0.5022	0.37589	1	0.5022	0.37589
3	0.49505	0.36774	2	0.29475	0.17346	2	0.49505	0.36774	2	0.36785	0.23676	2	0.3333	0.20575
3	0.55125	0.43405	2	0.73955	0.69385	2	0.5022	0.37589	2	0.45875	0.32766	2	0.32595	0.1994
3	0.45875	0.32766	3	0.2873	0.1675	2	0.73955	0.69385	2	0.2873	0.1675	2	0.2662	0.15111
3	0.73955	0.69385	3	0.2707	0.15454	2	0.92005	0.99734	2	0.2707	0.15454	2	0.34235	0.21368
3	0.51755	0.39367	3	0.49505	0.36774	2	0.66085	0.57821	4	0.55125	0.43405	2	0.73955	0.69385
3	0.32595	0.1994	3	0.92005	0.99734	2	0.36785	0.23676	4	0.9133	0.98503	3	0.49505	0.36774
3	0.8988	0.95884	3	0.66085	0.57821	2	0.1358	0.06598	4	0.6957	0.62817	3	0.9133	0.98503
												3	0.45875	0.32766
												3	0.6957	0.62817

HUVECIgG		HUVECIgA		HGECIgG		HGECIgA	
sanple133		sanple44	3.55587	sanple13	1.66779	sanple13	5.95427
*25	2.61769	*25	2.39672	*25	1.56023	*25	3.37622
*50	2.42293	*50	1.42694	*50	1.66351	*50	2.4304
*100	2.25831	*100	0.92005	*100	1.4985	*100	1.8218
*200	1.90975	*200	0.61018	*200	1.4215	*200	1.28105
*400	1.55114	*400	0.37299	*400	1.26994	*400	0.86201
*800	1.10906	*800	0.23145	*800	0.91181	*800	0.52479
*1600	0.77364	*1600	0.12433	*1600	0.71564	*1600	0.35319
*3200	0.49232	*3200	0.05547	*3200	0.40946	*3200	0.20553
*6400	0.30064	*6400	0.04591	*6400	0.28846	*6400	0.10376
*12800	0.18317	*12800	0.00191	*12800	0.24018	*12800	0.08381
blanc	0	blanc	0	blanc	0	blanc	0

HUVECIgG			HUVECIgA		HUGECIgG		HUGECIgA	
0.18317	0.01563	0.01605	0.00191	0.00781	0.24018	0.01563	0.08381	0.00781
0.30064	0.03125	0.01723	0.04591	0.01563	0.28846	0.03125	0.10376	0.01563
0.49232	0.0625		0.05547	0.03125	0.40946	0.0625	0.20553	0.03125
0.77364	0.125		0.12433	0.0625	0.71564	0.125	0.35319	0.0625
1.10906	0.25	0.2741	0.23145	0.125	0.91181	0.25	0.52479	0.125
1.55114	0.5		0.37299	0.25	1.26994	0.5	0.86201	0.25
1.90975	1		0.61018	0.5	1.4215	1	1.28105	0.5
			0.92005	1			1.8218	1

		Before Tx	After Tx
KID119	HUVECIg	1.008	0.538
	au huveci	0.2035	0.03699
	HUVECIg	0.3825	0.137
	au huveci	0.25049	0.06664
KID120	HUVECIg	1.179	0.225
	au huveci	0.3017	0.01021
	HUVECIg	0.691	0.0845
	au huveci	0.62132	0.04012
KID121	HUVECIg	0.344	0.377
	au huveci	0.01247	0.01481
	HUVECIg	0.145	0.146
	au huveci	0.07108	0.07164
KID133/1:	HUVECIg	1.91	0.519
	au huveci	0.94779	0.03344
	HUVECIg	0.913	0.162
	au huveci	0.98449	0.08085

KID120

no	KID	IgA-RE10(	cut off	Age	Gender	UP	UP g/gCr	UOB	URBC v	Alb	BUN	sCr	eGFR	IgG	IgA	ANA	C3	anti-DNA	PSL	TG	NIH activit	endocapill	score	fibrinoid r	karyorrhe	score(×2)	wire loop	hyaline th	score	cressent	score(×2)	Active+/-	IgA	IgA数値	IgG	IgG数値	C3	C3数値
42	KID126	0.06205	0	37	0	3	6.25	0	0	2.8	10.7	0.52	104	674	169	80	69	51.7	10	53	0	0	0	0	0	0	0	0	0	0	0	0	+	1	+	1	+	1
43	KID127	0.0975	0	30	1	3	2.59	0.5	1	3.4	11.5	0.74	101	1663	487	40	42	7.9	0	45	0	0	0	0	0	0	0	0	0	0	0	0	+	1	+	1	+	1
32	KID100	0.1022	0	33	0	3	4.076	2	6	3	20	0.9	59	448	103		82	67	15	23	0	0	0	0	0	0	0	0	0	0	0	0	-	0	+	1	+	1
47	KID132	0.1109	0	26	0	3	3.34	0	3	2.9	5.3	0.42	145.4	943	188	320	58	79	22.5	34	0	0	0	0	0	0	0	0	0	0	0	0	-	0	-	0	-	0
34	KID102	0.13045	0	32	1	2	2.141	2	6	2.9	8	0.6	125.5	4271	159	320	50	8	0	57	0	0	0	0	0	0	0	0	0	0	0	0	+	1	+	1	+	1
20	KID139	0.1358	0	43	0	3	2.256	3	35	4.1	14.4	0.53	97.6	1230	279	320	64	3	12.5	40	5	1	1	0	1	2	0	0	0	2	2	1	+	1	+	1	+	1
39	KID114	0.13815	0	25	0	2	1.387	2	15	3.2	9.5	0.51	121.8	3257	132	320	103	35	20	47	0	0	0	0	0	0	0	0	0	0	0	0	+	1	+	1	+	1
27	KID41	0.14475	0	26	0	3	4.034	0	3	2.9	9.9	0.67	87.2	988	136		55	12	9	83	0	0	0	0	0	0	0	0	0	0	0	0	+	1	+	1	+	1
25	KID39	0.14925	0	42	0	3	6.666	0.5	3	1.9	43.5	1.25	39.2	540	330	80	80	0	20	23	0	0	0	0	0	0	0	0	0	0	0	0	-	0	+	1	+	1
31	KID099	0.15655	0	64	0	2	2.615	0	0	3.7	14	0.7	64.2	910	282	20	70	19	11		0	0	0	0	0	0	0	0	0	0	0	0						
46	KID131	0.1618	0	24	0	0	0.03	0	1	4.2	8.7	0.52	117.8			80	93	30	10	25	0	0	0	0	0	0	0	0	0	0	0	0	+	1	+	1	+	1
38	KID111	0.1816	0	45	1	0	0.27	2	6	3.5	17.7	0.79	85.9	1370	334	20	55	0	12	10	0	0	0	0	0	0	0	0	0	0	0	0	+	1	+	1	+	1
33	KID101	0.18655	0	45	0	2	0.394	0	1	3.5	16	0.6	84.1	1802	358	40	43	25.3	6	15	0	0	0	0	0	0	0	0	0	0	0	0	+	1	+	1	+	1
5	KID104	0.1974	0	31	0	1	0.89	1	1	3.3	9	0.7	79	1014	319	80	60	41	20	17	4	3	1	0	0	0	2	0	1	1	2	1	+	1	+	1	+	1
29	KID046	0.19865	0	64	0	2	5.804	1	1	2.9	19.9	0.81	54.7	1499	284		45	23	5	16	0	0	0	0	0	0	0	0	0	0	0	0						
41	KID123	0.2028	0	20	0	3	2.163	0	3	3.7	15	0.6	109.4	300	55		68	0	5	23	0	0	0	0	0	0	0	0	0	0	0	0						
36	KID105	0.20365	0	48	0	3	7.76	0.5	1	1.9	19	0.7	69.7	2155	187	320	44	2	6	21	0	0	0	0	0	0	0	0	0	0	0	0	+	1	+	1	+	1
35	KID103	0.2199	0	36	0	3	4.271	1	6	2.1	15	0.9	57.5	526	216	80	111	0	6	27	0	0	0	0	0	0	0	0	0	0	0	0	+	1	+	1	+	1
51	RID-19	0.23705	0	64	0	0		0	3	4.5	17.6	0.63	72	2152	237		62	57		22	0	0	0	0	0	0	0	0	0	0	0	0						
40	KID116	0.24155	0	42	1	1	1.207	2	3	1.3	6.9	0.5	104.7	1749	346		35	68	20	22	0	0	0	0	0	0	0	0	0	0	0	0	+	1	+	1	+	1
49	KID140	0.24705	0	45	0	3	11.272	0.5	3	2.5	19.3	0.97	49.7	697	423	160	71	5.9	10	36	0	0	0	0	0	0	0	0	0	0	0	0	+	1	+	1	+	1
10	KID113	0.2662	0	65	0	2	2.817	3	6	3.6	19.6	0.83	53.8	838	246		122	2.8	20	39	4	11	2	0	0	0	0	0	0	1	2	1	+	1	+	1	+	1
14	KID119	0.2707	0	31	0	2	4.06	2	1	3.3	12.7	0.72	78.1	882	237	320	57	58	10	62	10	45	3	0	2	2	45	6	3	11	2	1						
6	KID106	0.2873	0	33	0	2	1.24	2	6	2.8	13	0.51	109.8	2170	199	160	24	28	5	7	8	4	3	0	0	0	3	1	3	1	2	1	+	1	+	1	+	1
16	KID122	0.29475	0	45	0	3	3.501	0.5	0	3.3	27.5	1.12	43	942	297	20	60	4.8	10	18	3	3	1	0	0	0	3	5	2	0	0	1	+	1	+	1	+	1
48	KID136	0.29895	0	35	0	3	2.413	0	0	3.4	12.6	0.56	97.4	1081	147	320	72	6.2	20	22	0	0	0	0	0	0	0	0	0	0	0	0	-	0	+	1	+	1
44	KID129	0.29915	0	23	0	0	0.88	2	25	3.5	10.8	0.44	143.1	1054	310	80	53	91	22.5	19	0	0	0	0	0	0	0	0	0	0	0	0	+	1	ー	0	2+	2
8	KID110	0.32595	0	35	0	3	4.237	3	100	2.4	21.1	0.8	67.7	1342	216	320	19	180	0	35	5	18	3	0	0	0	5	6	2	0	0	1	+	1	+	1	+	1
50	RID-04	0.3322	0	34	1	3	3.914	0	15	2.6	9.5	0.82	87.6	2049	248		28	0	15	38	0	0	0	0	0	0	0	0	0	0	0	0	+	1	+	1	+	1
7	KID107	0.3333	0	32	0	3	7.52	2	6	2.2	7	0.61	93.7	899	291	160	53	27	10	21	4	6	2	0	0	0	9	0	2	0	0	1	+	1	+	1	+	1
13	KID118	0.34235	0	39	0	2	0.986	3	9	2.9	15.5	0.72	72.9	1726	360	160	19	300	30	28	8	23	3	4	2	2	1	1	1	1	2	1	+	1	-	0	+	1
30	KID048	0.3464	0	41	0	3	7.252	0.5	3	4	12.9	0.42	128.5	904	256		78	4	8	32	0	0	0	0	0	0	0	0	0	0	0	0	+	1	+	1	+	1
19	KID138	0.36785	0	60	1	2	0.347	3	25	4.3	27.2	1.02	58.6	1172	284	320	54	11	5	19	5	0	0	1	0	2	0	2	1	2	2	1	+	1	+	1	2+	2
	quaritile1	0.14925	0	31	0	2	1.23175	0	1	2.8	9.9	0.53	64.2	894.75	187.75	80	45	4	6	20.5	0	0	0	0	0	0	0	0	0	0	0	0		1		1		1
	quaritile3	0.29475	0	45	0	3	4.2455	2	6	3.5	19	0.81	104.7	1731.75	312.25	320	71	51.7	20	38.25	4	1	1	0	0	0	0	0	0	0	0	0		1		1		1
	median	0.20365	0	36	0	3	2.716	1	3	3.2	14	0.7	85.9	1067.5	252	160	58	19	10	24	0	0	0	0	0	0	0	0	0	0	0	0		1		1		1
	average	0.22032	0	39.2424	0.18182	2.21212	3.39322	1.18182	9	3.10606	15.1606	0.70091	86.7758	1351.47	253.594	172.5	60.5758	37.8061	11.7344	30.5938	1.69697	3.45455	0.57576	0.15152	0.15152	0.24242	2.06061	0.63636	0.45455	0.57576	0.42424	10		24		25		25
	strev	0.08441	0	12.5972	0.39167	1.0234	2.6574	1.10975	18.2551	0.74159	7.45352	0.20093	28.5004	827.751	93.9584	123.825	24.2436	60.0853	7.47143	16.4255	2.87755	9.14498	1.06155	0.71244	0.50752	0.66287	7.93308	1.67366	0.90453	1.95305	0.8303	23		4		3		1
17	KID125	0.41005	1	75	0	3	4.35	2	6	2.7	45.3	0.97	43.1	1901	581	320	52	12	40	36	2	2	1	0	0	0	3	0	1	0	0	1	+	1	+	1	+	1
26	KID40	0.447	1	29	0	3	2.491	0	0	3.9	11.1	0.5	118.8	2625	377	320	66	37	0	36	0	0	0	0	0	0	0	0	0	0	0	0	+	1	+	1	+	1
22	KID142	0.45875	1	42	0	3	6.119	3	100	2.4	19.9	0.65	79.1	1562	360	160	24	100	20	34	7	27	3	0	0	0	9	5	2	1	2	1	+	1	+	1	+	1
28	KID045	0.47445	1	42	0	2	1.255	1	3	3.3	9.7	0.44	121.2	3492	307	320	43	8.8	0	20	0	0	0	0	0	0	0	0	0	0	0	0	+	1	+	1	+	1
4	KID047	0.49505	1	28	0	3	1.015	3	100	3.1	15.6	0.76	74.4	1248	167	320	19	300	30	45	8	32	3	3	5	2	36	0	3	0	0	1	+	1	+	1	+	1
24	KID144	0.5022	1	25	0	1	0.595	1	3	3.4	13.2	0.48	127	3288	182	320	22	300	0	97	7	29	2	14	1	2	0	9	1	8	2	1	+	1	+	1	+	1
1	KID042	0.51755	1	40	1	3	3.25	3	35	2.4	18.2	0.96	71.4	1694	413	80	46	300	5	26	5	20	3	0	0	0	0	0	0	1	2	1	+	1	-	0	+	1
11	KID115	0.5225	1	38	0	1	0.472	2	15	3.2	21.4	0.8	66	1504	384	160	48	65	9	38	2	1	1	0	0	0	1	0	1	0	0	1	+	1	+	1	+	1
45	KID130	0.52705	1	17	0	3	3.46	3	25	3.1	22.5	0.63	105	812	120		39	35	35	18	0	0	0	0	0	0	0	0	0	0	0	0	+	1	+	1	+	1
12	KID117	0.55125	1	63	0	3	6.113	3	1	2.4	18	0.85	52.6	1556	193	320	84	130	0	25	7	18	3	0	0	0	0	0	0	10	4	1	+	1	+	1	+	1
9	KID112	0.58135	1	37	0	1	0.512	0.5	6	3.8	12.3	0.74	72.4	1586	373	40	49	51	12	38	4	11	2	0	0	0	0	0	0	1	2	1	+	1	+	1	+	1
37	KID109	0.61695	1	20	0	3	3.014	1	15	2.1	23.9	0.78	79.6	1808	255	320	40	110	40	13	0	0	0	0	0	0	0	0	0	0	0	0	+	1	+	1	+	1
2	KID043	0.66085	1	28	0	1	1.451	2	1	3.5	10.1	0.63	93.3	1530	161	320	19	300	20	58	6	13	1	1	0	2	36	13	3	0	0	1	+	1	+	1	+	1
23	KID143	0.6957	1	64	0	3	6.152	3	100	2.7	19.5	0.95	46	1959	284	320	45	67	0	15	10	9	3	1	1	2	0	1	1	4	4	1						
21	KID140	0.73955	1	38	0	2	0.606	2	1	3.1	11.4	0.59	89.9	2351	335	320	14	300	20	50	7	31	3	1	1	2	4	14	2	0	0	1	+	1	+	1	+	1
15	KID120	0.8988	1	30	0	3	9.286	3	100	1.9	19.2	1.1	49.6	2343	254	320	19	300	0	42	4	34	3	0	0	0	1	0	1	0	0	1	+	1	+	1	+	1
18	KID133	0.9133	1	16	0	3	4.6	3	25	2.3	33.1	1.56	39.7	2817	154	320	26	300	0	35	10	26	3	2	4	2	3	2	1	14	4	1	+	1	2+	2	+	1
3	KID044	0.92005	1	24	0	1	0.368	3	1	3.5	16.7	0.74	81	1790	247	320	41	300	20	15	6	2	1	1	0	2	4	4	3	0	0	1	+	1	+	1	+	1
	quaritile1	0.49684	1	25.75	0	1.25	0.70825	1.25	1.5	2.4	12.525	0.63	55.95	1557.5	184.75	320	22.5	54.5	0	21.25	2	1.25	1	0	0	0	0	0	0	0	0	0		1		1	#NUM!	1
	quaritile3	0.68699	1	41.5	0	3	4.5375	3	32.5	3.375	21.025	0.925	92.45	2349	369.75	320	47.5	300	20	41	7	26.75	3	1	0.75	2	3.75	3.5	1.75	1	2	0		1		1	#NUM!	1
	median	0.53915	1	33.5	0	3	2.7525	2.5	10.5	3.1	18.1	0.75	76.75	1799	269.5	320	40.5	120	10.5	35.5	5.5	12	2	0	0	0	0.5	0	1	0	0	0		1		1	#NUM!	1
	average	0.60736	1	36.4444	0.05556	2.33333	3.06161	2.13889	29.8333	2.93333	18.95	0.785	78.3389	1992.56	285.944	270.588	38.6667	167.544	13.9444	35.6111	4.72222	14.1667	1.77778	1.27778	0.66667	0.77778	5.38889	2.66667	1.05556	2.16667	1.11111	14		17		15	0	17
	strev	0.16417	0	16.4896	0.2357	0.90749	2.59098	1.02621	39.8516	0.5921	8.79995	0.26553	27.0509	701.813	117.49	95.4247	18.3367	125.522	14.7028	19.912	3.39213	13.0124	1.26284	3.28643	1.45521	1.00326	11.3818	4.62728	1.10997	4.16215	1.56765	4		0		1	0	0

V4 の %	1	1	1

カイ 2 乗検定

	値	自由度	漸近有意確率 (両側)
Pearson の	1.92621	2	0.38171
尤度比	2.24318	2	0.32576
線型と線型	1.1131	1	0.29141
有効なケー	45		

対称性による類似度

		値	近似有意確率
名義と名義	ファイ	0.20689	0.38171
	Cramer の	0.20689	0.38171
	分割係数	0.2026	0.38171
有効なケースの数		45	

IgA数値 * V4

クロス表

			V4 0	V4 1	合計
IgA数値	0	度数	4	0	4
		V4 の %	0.14286	0	0.08889
		調整済み残	1.63264	-1.63264	
	1	度数	24	17	41
		V4 の %	0.85714	1	0.91111
		調整済み残	-1.63264	1.63264	
合計		度数	28	17	45
		V4 の %	1	1	1

カイ 2 乗検定

	値	自由度	漸近有意確	正確有意確	正確有意確率 (片側)
Pearson の	2.66551	1	0.10255		
連続修正b	1.1934	1	0.27465		
尤度比	4.02985	1	0.0447		
Fisher の直接法				0.28118	0.13742
線型と線型	2.60627	1	0.10644		
有効なケー	45				

対称性による類似度

		値	近似有意確率
名義と名義	ファイ	0.24338	0.10255
	Cramer の	0.24338	0.10255
	分割係数	0.23648	0.10255
有効なケースの数		45	
