# Supplementary figures and images for: The Level of IgA Antibodies to Endothelial Cells Correlates with Histological Evidence of Disease Activity in Patients with Lupus Nephritis
Source: PLoS One. 2016 Oct 27;11(10):e0163085. doi: 10.1371/journal.pone.0163085 (PMC5082850; doi:10.1371/journal.pone.0163085)

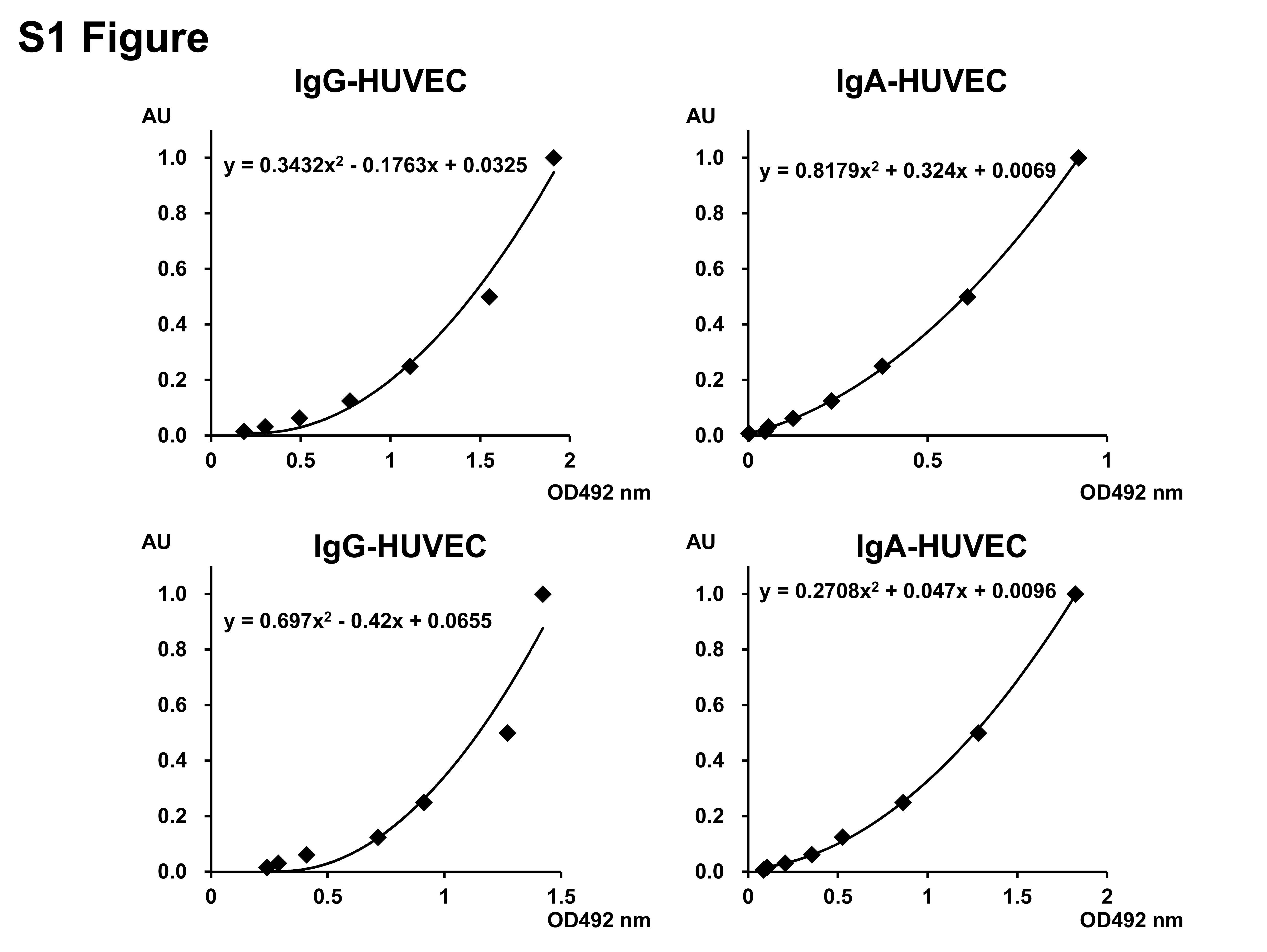

Supplement: S1 Fig — Standard curves of CSP-ELISA for IgG-HUVEC, IgA-HUVEC, IgG-HGEC, and IgA-HGEC were generated from serum showing the highest OD 492 nm values. Second-order polynomial trend lines were produced best fit with the data. (TIF) [file pone.0163085.s002.tif]

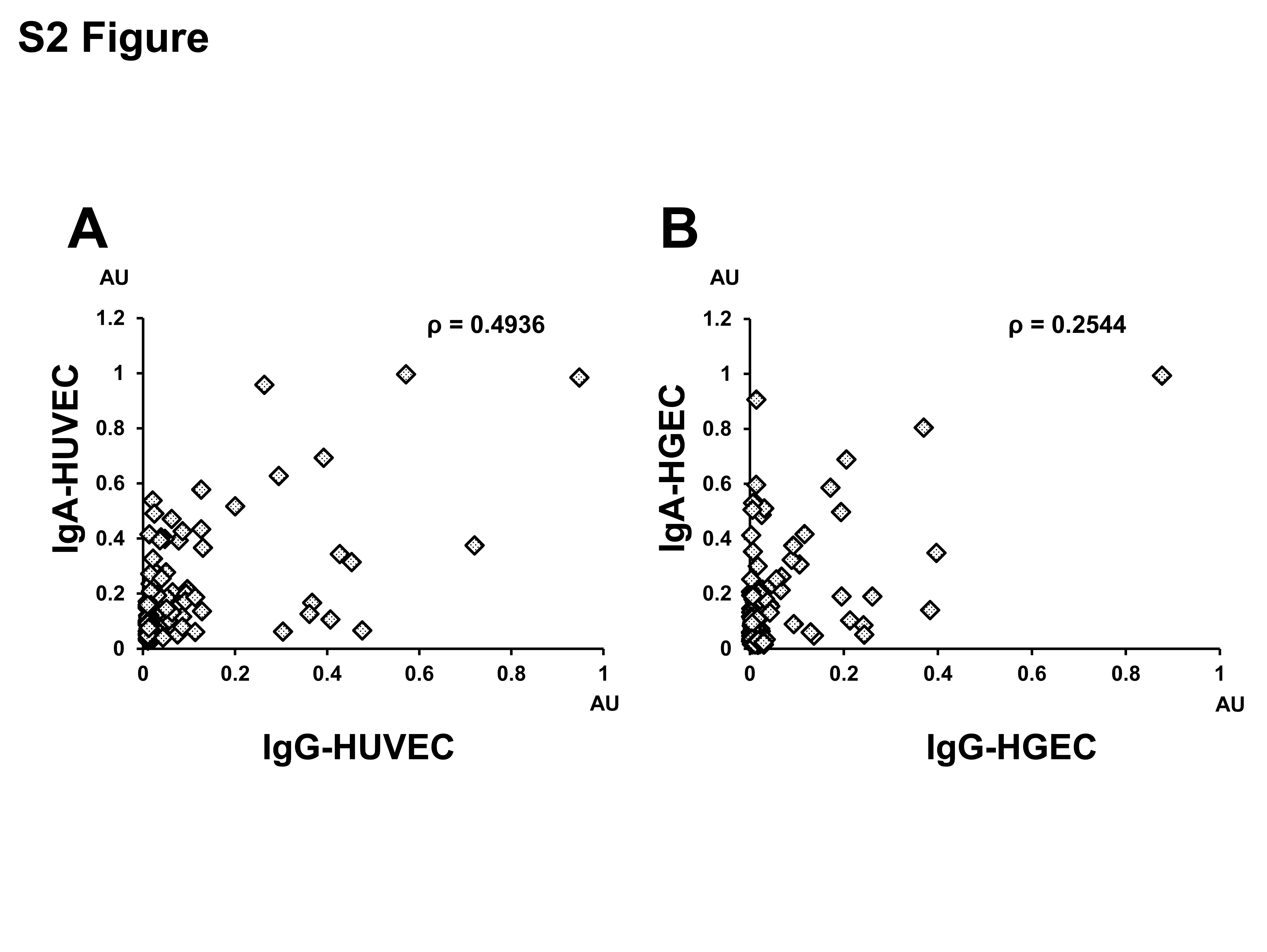

Supplement: S2 Fig — A. The level of IgG- and IgA-HUVEC is not correlated well. B. The level of IgG- and IgA-HGEC is not correlated well. (TIF) [file pone.0163085.s003.tif]

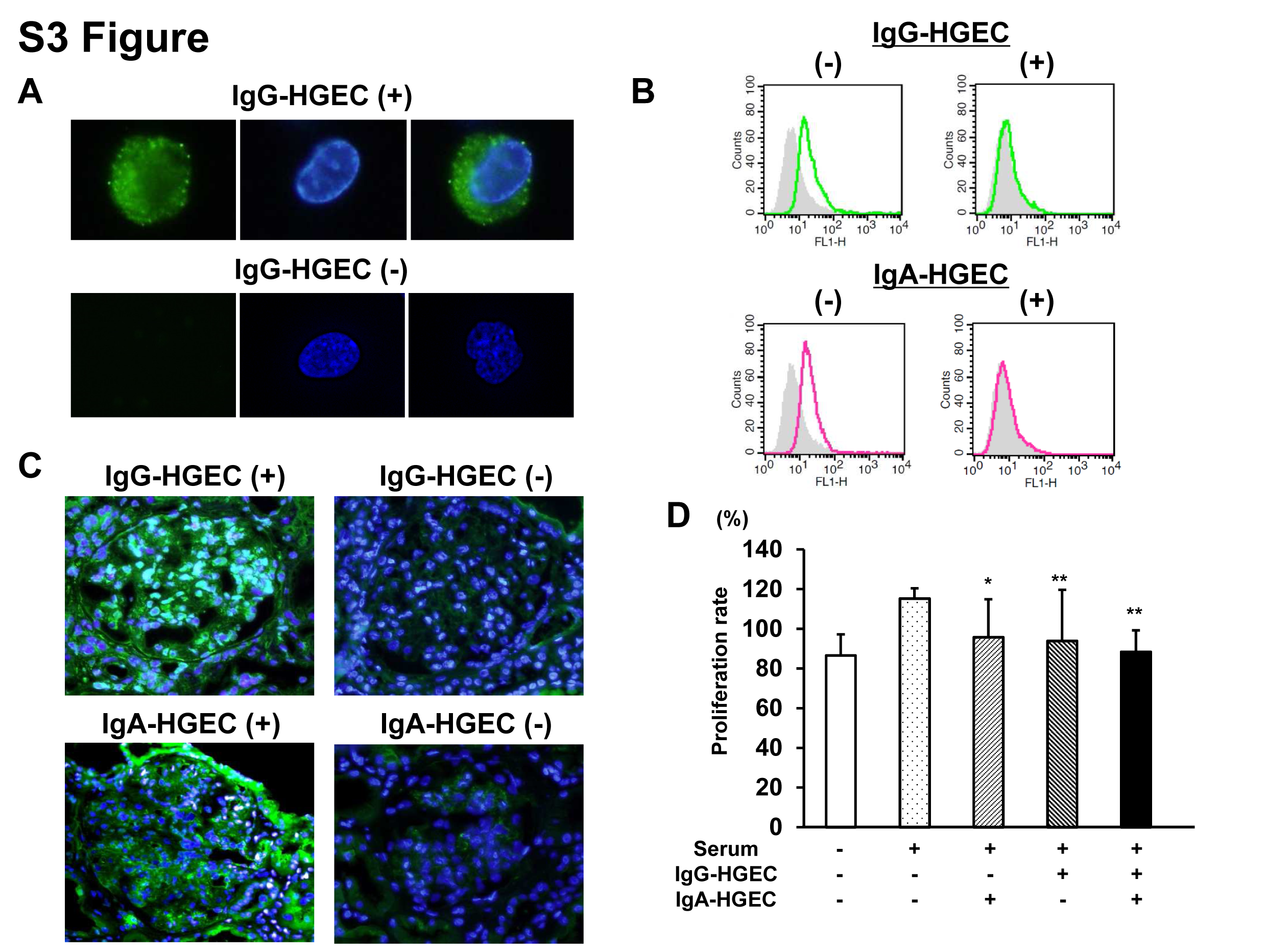

Supplement: S3 Fig — A. Immunocytochemical analysis showed positive staining in HGEC for IgG using serum from LN patients with high titer of AECA. B. Flow cytometric analysis using serum of LN patients demonstrated that IgG- or IgA-AECA bind strongly to HGEC. C. Serum IgG- and IgA-AECA to glomerular endothelial cells were detected via immunofluorescence on normal renal tissue cryosections. D. The proliferation of HGEC incubated with serum containing high titer of IgG or IgA-AECA were significantly decreased than those without AECA. *P < 0.05, **P < 0.01 vs IgA and IgG negative serum (Scheffe test). (TIF) [file pone.0163085.s004.tif]

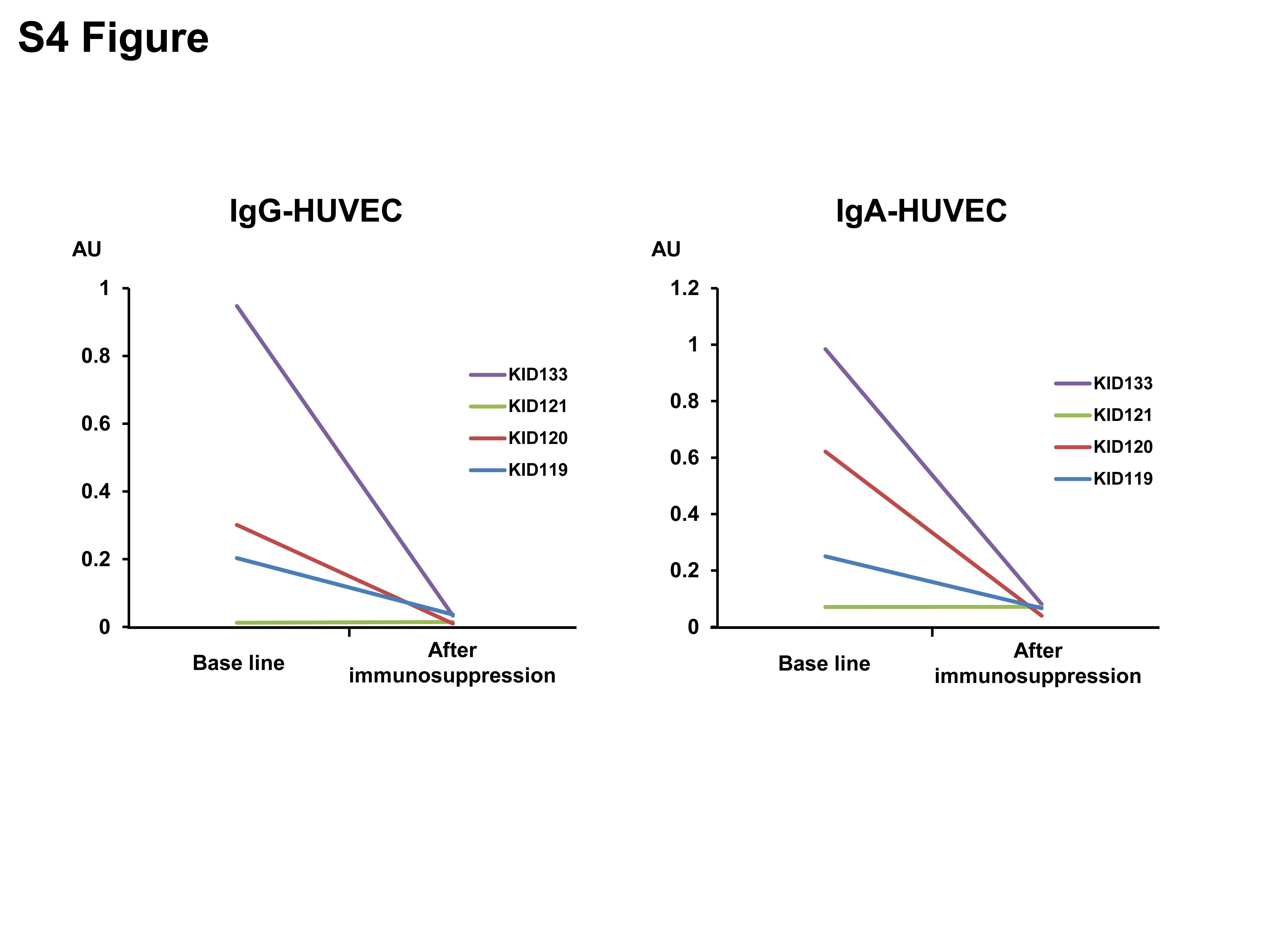

Supplement: S4 Fig — (TIF) [file pone.0163085.s005.tif]
